# The Methylation Game: Epigenetic and Epitranscriptomic Dynamics of 5-Methylcytosine

**DOI:** 10.3389/fcell.2022.915685

**Published:** 2022-06-03

**Authors:** Adele Alagia, Monika Gullerova

**Affiliations:** Sir William Dunn School of Pathology, University of Oxford, Oxford, United Kingdom

**Keywords:** DNA, RNA, m^5^C, hm^5^C, methyltransferases, demethylases, cancer, DNA damage

## Abstract

DNA and RNA methylation dynamics have been linked to a variety of cellular processes such as development, differentiation, and the maintenance of genome integrity. The correct deposition and removal of methylated cytosine and its oxidized analogues is pivotal for cellular homeostasis, rapid responses to exogenous stimuli, and regulated gene expression. Uncoordinated expression of DNA/RNA methyltransferases and demethylase enzymes has been linked to genome instability and consequently to cancer progression. Furthermore, accumulating evidence indicates that post-transcriptional DNA/RNA modifications are important features in DNA/RNA function, regulating the timely recruitment of modification-specific reader proteins. Understanding the biological processes that lead to tumorigenesis or somatic reprogramming has attracted a lot of attention from the scientific community. This work has revealed extensive crosstalk between epigenetic and epitranscriptomic pathways, adding a new layer of complexity to our understanding of cellular programming and responses to environmental cues. One of the key modifications, m^5^C, has been identified as a contributor to regulation of the DNA damage response (DDR). However, the various mechanisms of dynamic m^5^C deposition and removal, and the role m^5^C plays within the cell, remains to be fully understood.

## Introduction

Gene expression regulation is not only affected by mutations in the genomic sequence, but also by various other molecular mechanisms (Allis and Jenuwein, 2016), such as epigenetic regulation. Epigenetic regulation can influence gene expression profiles regardless of the genomic sequence, and can result in either the condensation or relaxation of the chromatin structure (Dai et al., 2020). The main players in epigenetic control; m^5^C DNA methylation, histone modifications, and non-coding RNAs (Kumar et al., 2018), have been linked to genome stability and are known to contribute to the maintenance of genome integrity (Deem et al., 2012). Three distinct aspects govern epigenetic status; inheritance, environmental factors, and stability over time (Bonasio et al., 2010). Epigenetic modifications are stable enough to maintain cellular homeostasis, but are also reversible to allow transitions among different states and responses to environmental cues (Zhang et al., 2019). The dynamic nature of epigenetic features controls several aspects of transcription regulation and consequently influences many physiological processes, such as development (John and Rougeulle, 2018), transposon control (Misiak et al., 2019), and brain and memory formation (Kim and Kaang, 2017). Furthermore, deregulation of epigenetic processes can lead to genomic instability, promoting the onset and development of different diseases including cancer (Lu et al., 2020) (Liu et al., 2017). For example, during cancer development, genome-wide DNA hypomethylation and gene-specific hypermethylation occur as a consequence of mutated or deregulated chromatin modifiers (Ibrahim et al., 2022). Thus, epigenetic alterations or epimutations (McCarrey et al., 2016) result in abnormal transcriptional repression or activation of genes (Lee, 2019). Epimutations can be classified into two groups: primary, which are epigenetic alterations in the absence of a genetic change, and secondary epimutations, which are acquired as a consequence of DNA mutations in genes of *cis* or *trans*-acting chromatin factors (Oey and Whitelaw, 2014). Interestingly, secondary epimutations are the most reported in cancers (Ruiz de la Cruz et al., 2021). At a molecular level, epigenetics involves a complex and dynamically reversible set of structural modifications of chromatin catalysed by enzymes often referred to as “writers,” which add different chemical modifications such as methyl group moieties to DNA (Biswas and Rao, 2018). These molecular decorations are then able to recruit a plethora of proteins called “readers” that specifically recognise these moieties (Du et al., 2015). Finally, a set of “erasers” catalyses the removal of the deposited modification. Writers, readers, and erasers function dynamically to regulate the epigenetic landscape. Concerted variations of epigenetic modifications ensure an organism’s normal development and its responsiveness to environmental stimuli (Norouzitallab et al., 2019). Removal and restoration of methylation marks is also important during embryonic development, with coordinated waves of demethylation and *de novo* methylation establishing specific cell fates (Wu and Zhang, 2014). With recent advances in high-throughput sequencing techniques and transcriptome-wide studies, more than 150 post-transcriptional modifications have been also described on RNA, termed the epitranscriptome (Ma et al., 2022). Similarly to DNA methylation, RNA bases can be methylated and can function in the fine-tuning of gene expression (Kumar and Mohapatra, 2021) (Seo and Kleiner, 2021) (Willbanks et al., 2021). Epitranscriptomic studies have also revealed how post-transcriptional RNA modifications can dynamically affect several aspects of RNA metabolism including processing, export, translation, and RNA stability (Flamand and Meyer, 2019; Ranjan and Leidel, 2019; Trixl and Lusser, 2019; Boo and Kim, 2020; Chen et al., 2021a; Kumar and Mohapatra, 2021; Schaefer, 2021). Furthermore, epitranscriptomic changes have been demonstrated to play a crucial role in stress response processes (such as the DNA damage response) (Jimeno et al., 2021; Wilkinson et al., 2021) and aberrant epitranscriptomes are associated with several human diseases, including cancer (Hsu et al., 2017; Jiang et al., 2017; Lian et al., 2018; Esteve-Puig et al., 2020). Unlike epigenetic DNA modifications, RNA methylation cannot be transferred into offspring and can result in significant changes in RNA secondary structure (Schaefer et al., 2017). Changes in base-pairing potential and protein-RNA interactions make epitranscriptomics a complex cellular mechanism that impacts both RNA metabolism and gene expression (Kan et al., 2022). Epigenetic and epitranscriptomic marks have the ability to expand the physicochemical features of the A-T-C-G nucleobases. The m^5^C DNA modification, known as the fifth base, has been extensively described as a CpG-specific modification able to modulate chromatin architecture with the assistance of repressive histone mark deposition. More recently, significant technical progress for RNA m^5^C detection approaches has been made (Yuan et al., 2019). Novel techniques, including m^5^C RNA immunoprecipitation sequencing (m^5^C-RIP-seq), 5-AZA-cytidine-mediated RNA immunoprecipitation sequencing (Aza-IP-seq) (Guo et al., 2021), methylation-individual nucleotide resolution crosslinking immunoprecipitation sequencing (miCLIP-seq) (Chen et al., 2021a) and TET-assisted peroxotungstate oxidation sequencing (TAWO-seq) (Yuan et al., 2019), were successfully developed and applied. The presence of m^5^C has been detected in diverse RNA molecules including tRNAs, rRNAs, mRNAs, viral RNAs, and ncRNAs (George et al., 2017; Genenncher et al., 2018; Zeng et al., 2018; García-Vílchez et al., 2019; Trixl and Lusser, 2019; Zhao et al., 2020). Although the m^5^C modification on RNA has been demonstrated to play a role in the pathogenesis of several diseases, the mechanism of action remains largely unexplored. Furthermore, evidence of changes in the transcriptional landscape driven by m^5^C during several physiopathological processes, ranging from pluripotency, development, differentiation, genome instability, and oncogenesis have been reported (Frye et al., 2018; Bohnsack et al., 2019; He et al., 2020; Song et al., 2022). A large amount of scientific literature describes cancer as a genetic, epigenetic, and epitranscriptomic disease (Esteller and Pandolfi, 2017; Lobo et al., 2018; Porcellini et al., 2018; Xue et al., 2020a; Esteve-Puig et al., 2020; Xie et al., 2020; Miano et al., 2021; Lopez et al., 2022). Oncogenesis driven by genome instability and consequently accumulation of mutations is a complex pathological process that involves changes in gene expression. Both hypermethylation and hypomethylation at different genetic loci and of RNAs is strongly correlated to tumour initiation, progression, and metastasis (Pérez et al., 2018; Locke et al., 2019; Nishiyama and Nakanishi, 2021). Furthermore, deregulated deposition and removal of methylation marks as a result of deregulated writer and eraser enzymes has also been described as a hallmark of cancer chemotherapy resistance (Ginno et al., 2020; Romero-Garcia et al., 2020; Feng et al., 2021a; Sun et al., 2021a; Zhao et al., 2021). In this review, we summarise the current insights surrounding the dynamics of m^5^C and its oxidized derivatives (hm^5^C, f^5^C, and ca^5^C) and their relevance in patho-physiological contexts such as pluripotency, development, differentiation, the DNA damage response, and cancer.

## DNA/RNA Cytosine Methylation

**FIGURE 1:**
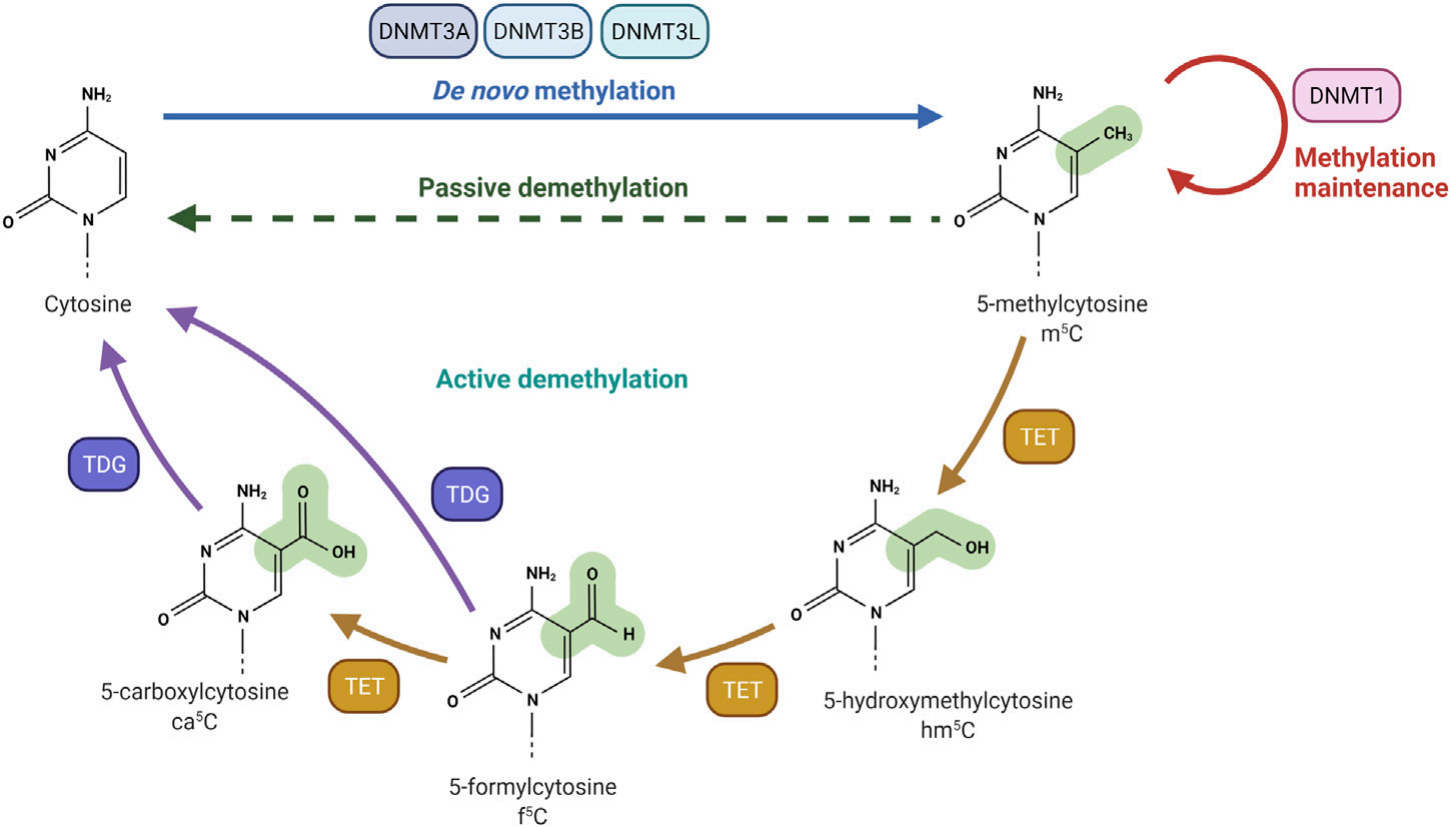
DNA methylation and demethylation machinery. DNA methyltransferase 3A (DNMT3A) and DNA methyltransferase 3B (DNMT3B) are responsible for the creation of DNA methylation patterns. DNA methyltransferase 3L (DNMT3L) interacts and stimulates DNMT3A and DNMT3B methylation activity. DNA methyltransferase 1 (DNMT1) is actively involved in the maintenance of DNA methylation patterns. Passive demethylation occurs in the absence of functional DNMT1, which methylates DNA after each cellular division. Active demethylation is mediated by Ten-eleven Translocation (TET) dioxygenases. TET enzymes oxidize 5-methylcytosine (m^5^C) to produce 5-hydroxymethylcytosine (hm^5^C), 5-formylcytosine (f^5^C) and 5-carboxylcytosine (ca^5^C). The glycosylase activity of TDG allows the excision of Tet-produced f^5^C or ca^5^C nucleobases. Created with BioRender.com.

**FIGURE 2:**
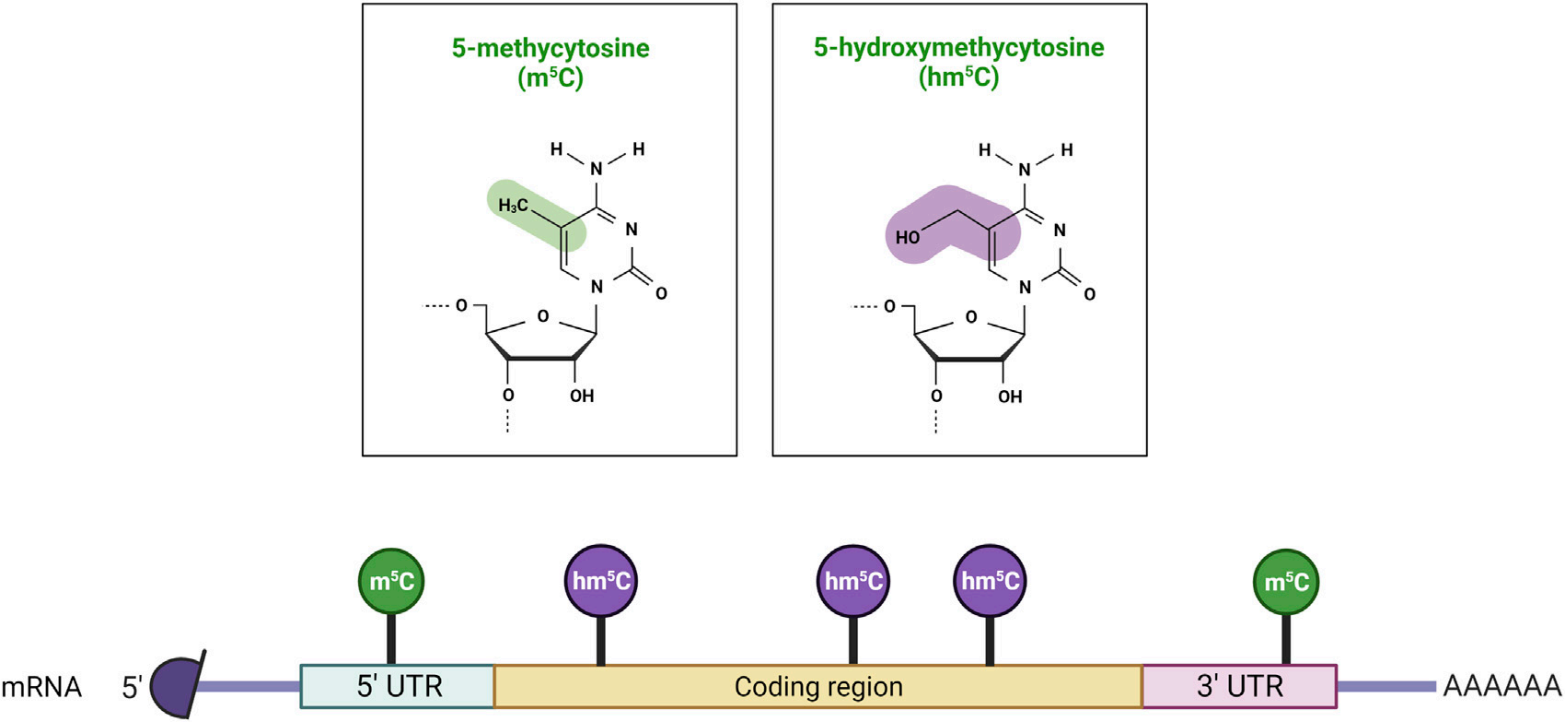
Distribution of m^5^C and hm^5^C post-transcriptional modifications on mRNA molecules. 5-methylcytosine (m^5^C) is predominantly found in 5′ untranslated regions (5′ UTRs), while 5-hydroxymethylcytosine (hm^5^C) is present within the coding sequence (CDS). Created with BioRender.com.

The addition of a methyl group at the carbon-5 position of cytosine in DNA in the CpG dinucleotide context is catalysed by the S-adenosyl-methionine-dependent DNMT methyltransferase family. DNMT1 primarily maintains DNA methylation patterns during replication, while DNMT3A, DNMT3B, and DNMT3L are predominantly involved in the establishment of *de novo* DNA methylation (Figure 1) (Lyko, 2018). Proper maintenance of DNA methylation patterns defines the structural and functional identities of cells throughout cell division (Moore et al., 2013). Although DNA methylation patterns are stable, active and passive demethylation modulates a dynamic methylation process (Sadakierska-Chudy et al., 2015). Passive demethylation or replication-dependent dilution occurs after the synthesis of newly replicated DNA strands. Without functional DNA m^5^C methylation maintenance of newly synthesized DNA strands, the symmetry of methylation is not re-established and methylation is lost through replication cycles. On the other hand, DNA methylation can be actively reversed by family of α-ketoglutarate-dependent dioxygenases, known as Ten-Eleven Translocation (TET) proteins, which exist as 3 isoforms: TET1, TET2, TET3 (Lan et al., 2020). Hydroxylation of m^5^C analogue to 5-Hydroxymethylcytosine (hm^5^C), alters the affinity of DNMT1 for the methylated site, and results in loss of the epigenetic mark over several rounds of DNA replication. RNA m^5^C modification has been found in mRNAs, rRNAs, tRNAs, and ncRNAs. The RNA specific subset of S-adenosyl-methionine-dependent methyltransferases includes TRDMT1 (tRNA aspartic acid methyltransferase 1) (Li et al., 2021a), also known as DNMT2, and the NSUN1-7 (NOP2/Sun RNA methyltransferases) family, which are responsible for the deposition of m^5^C on RNA (Bohnsack et al., 2019; Sun et al., 2019; Liu and Santi, 2000). The m^5^C mark is reported to be involved in the regulation of RNA metabolism and is principally associated with structural and functional RNA stability, and its dynamic deposition and removal also permits rapid cellular responses to environmental cues (Gkatza et al., 2019). For example, several lines of evidence identified the m^5^C modification as a modulator of the maturation, stability, and translation of mRNA molecules, and is also important for nuclear export (Schumann et al., 2020; Huang et al., 2019). Changes in the m^5^C deposition pattern in mRNA is also associated with several hallmarks of cancer including cell survival, proliferation, invasion, and resistance to therapy (Zhang et al., 2020a; Nombela et al., 2021; Zhang et al., 2021; Xue et al., 2020b). Furthermore, the majority of m^5^C patterns on RNA are lineage- and tissue-specific (Amort et al., 2017). The presence of the m^5^C modification has been described on both the small and large rRNA subunits. M^5^C controls ribosome synthesis and processing, and can alter the conformation of the rRNA, effecting translation fidelity (Schosserer et al., 2015; Popis et al., 2016). In tRNA, m^5^C is mostly present at the junction of the variable loop and the T-stem spanning positions (47–50) (Van Haute et al., 2019). The presence of m^5^C on tRNA has been linked to proper folding of the tRNA molecule into an L-shaped structure. However, m^5^C has been also shown to be present at the C38 position in the anticodon loop of tRNA and can modulate the translation fidelity of a specific subset of genes (Huang et al., 2021a). In mRNA, the m^5^C modification is enriched in 5′/3′-UTRs, next to Argonaute-binding regions, but is depleted in coding regions (Figure 2). m^5^C has been also detected in many ncRNAs such as lncRNAs, lincRNAs, pseudogene transcripts, antisense transcripts, and vault RNAs (Amort et al., 2013; Khoddami and Cairns, 2013; Sajini et al., 2019; Sun et al., 2020), and its presence is likely to be linked to processing, stability, and interaction with m^5^C reader proteins.

## 5-Methylcytosine Modification in Cancer Onset

m^5^C-driven epigenetic and epitranscriptomic events are entwined in cellular homeostasis. Deregulated m^5^C deposition can promote cancer development as a consequence of deregulation of both DNA and RNA molecules (Sun et al., 2021b). The m^5^C-mediated upregulation of proto-oncogenes or silencing of tumour suppressor genes, together with an enhanced translation rate and stability of mRNA oncogenes, are common molecular events in many types of cancers including leukaemia, breast, bladder, gastric, ovarian, colorectal, and lung (Atala, 2020; Zhang et al., 2020a; Sun et al., 2020; Hu et al., 2021a; Awah et al., 2021; Huang et al., 2021b; Li et al., 2021b). Furthermore, alterations in DNMTs expression levels or activity have been linked to both hypermethylation of tumour-suppressor genes and hypomethylation of proto-oncogene genes (Nishiyama and Nakanishi, 2021). In addition, overexpression of the RNA m^5^C writer NSUN2 (Okamoto et al., 2012; Yi et al., 2017; Cheng et al., 2018; Chellamuthu and Gray, 2020; Xiang et al., 2020; Hu et al., 2021a; Su et al., 2021; Wang et al., 2022) and RNA m^5^C reader YBX1, has been associated with mRNA hypermethylation and oncogene activation (Chen et al., 2019). Furthermore, the NSUN2-YBX1-oncogene axis is a commonly deregulated pathway during the cancer onset and progression (Wang et al., 2022).

## 5-Methylcytosine Modification in DNA Damage Response

Since several RNA species (DNA damage response RNAs, damage-induced lncRNAs, *de novo* transcripts and DNA:RNA hybrids) have been described to be essential during DNA damage repair (Ketley and Gullerova, 2020), a pioneering study has investigated the possibility of RNA post-transcriptional modification relevance in DNA double strand break (DSB) repair. DNMT2/TRDMT1 protein was shown to be recruited to damage sites, where it is able to catalyse the deposition of m^5^C residues onto DNA:RNA hybrids (Chen et al., 2020a). m^5^C-modified DNA:RNA hybrids promoted the recruitment of specific readers including Rad52, which drives the later stages of DNA repair. In addition, loss of DNMT2 proved to be detrimental during the DNA damage response, suggesting the importance of m^5^C presence during HR-mediated DNA damage repair (Zhu et al., 2021).

## Ten-Eleven Translocation-Driven Iterative 5-Methylcytosine Oxidation (5-Hydroxymethylcytosine > 5-Formylcytosine > 5-Carboxylcytosine) in DNA and RNA

The presence of m^5^C in the genome is dynamically controlled by the antagonising action of specific writers and erasers. DNMT family members are mainly responsible for the deposition of m^5^C at both the DNA and RNA level, while Ten-eleven translocation (TET) proteins are considered m^5^C erasers. Active demethylation of m^5^C is achieved by TET-mediated sequential oxidation with the production of hm^5^C (5-Hydroxymethylcytosine), f^5^C (5-formylcytosine), ca^5^C (5-carboxylcytosine) analogues (Figure 1) (Ito et al., 2011). Then, the N-glycosidic bond of f^5^C and ca^5^C is processed by the Thymine DNA Glycosylase (TDG) to form abasic sites, followed by Base Excision Repair (BER) to restore the unmodified cytosine (He et al., 2011; Bordin et al., 2021). Alternative pathways include; 1) direct deformylation (f^5^C) and decarboxylation (ca^5^C) mediated by DNMT3A/B (Feng et al., 2021b), 2) AID/APOBEC-dependent deamination and production of 5hmU followed by TDG cleavage (Cervantes-Gracia et al., 2021), and 3) passive DNA replication-dependent loss (Vincenzetti et al., 2019). However, TET enzymes appeared to be involved in the oxidation of both DNA and RNA m^5^C (Wu and Zhang, 2011; Fu et al., 2014; He et al., 2021a). *In vitro* studies confirmed that double stranded DNA is the preferred TET substrate, followed by DNA:RNA hybrids, single stranded DNA and single stranded RNA. Double strand RNA molecules were shown not to be TET substrates, likely due to TET discrimination against the RNA A-form conformation (DeNizio et al., 2019).

## 5-Hydroxymethylcytosine

The hm^5^C mark has been annotated at promoters, enhancers, and in gene bodies (Cui et al., 2020). While hm^5^C is mainly associated with active gene transcription and an open chromatin structure, its role depends on the genomic context (active vs. poised genes) (Choi et al., 2014). Furthermore, hm^5^C modification has a locus and tissue specific signature and serves as a feature of cellular state and identity. Genome-wide mapping at a single-nucleotide resolution level using modification specific antibodies, has estimated that around the 5% of cytosine residues in mammalian genome are modified as m^5^C and less than the 1% are hm^5^C (He et al., 2021b). The presence hm^5^C has been described not only at promoters, enhancers and gene body regions, but also on several RNA molecules (Delatte et al., 2016). m^5^C and hm^5^C modifications have been identified as stable epigenetic marks, and different chromatin-binding proteins have been shown to specifically bind to either m^5^C or hm^5^C, suggesting these modifications have distinct functions in epigenetic regulation. Recent studies have identified several proteins which preferentially “read” m^5^C or its oxidized forms. For example, the Methyl-CpG binding domain (MBD) protein family plays a pivotal role in determining the transcriptional state of the epigenome and shows a strong preference for hm^5^C over the m^5^C modification (Buchmuller et al., 2020). MBD proteins mainly belong to chromatin-bound repressor complexes, which coordinate crosstalk between m^5^C methylation, histone modifications, and chromatin organization. TET-mediated hm^5^C biogenesis blocks the reader function of the MBDs and alleviates their transcriptional repression, producing a new platform for hm^5^C specific readers and transcriptional activation.

## 5-Formylcytosine and 5-Carboxylcytosine

Despite the fact that f^5^C and ca^5^C oxidized forms of m^5^C are considered short-lived intermediates in the demethylation process, and their steady-state levels are many orders of magnitude lower than hm^5^C, emerging evidence indicates that f^5^C and ca^5^C might have independent epigenetic signalling roles in recruiting modification-specific “reader” proteins (Song and He, 2013). f^5^C and ca^5^C can be recognized by transcriptional regulators, DNA repair factors, and chromatin regulators, predominantly stimulating gene activation. Beside these regulatory roles, some studies have proposed the TET-mediated oxidative products f^5^C and ca^5^C are mutagenic bases that can threaten the genomic integrity if not properly eliminated. Moreover, deregulation of TET-TDG-BER pathway and inefficient f^5^C and ca^5^C clearance has been linked to DNA damage and the production of DSBs (Weber et al., 2016).

## 5-Hydroxymethylcytosine Modification in Pluripotency, Development, and Differentiation

Transcriptome flexibility is required for embryonic stem cell (ESC) differentiation (Dawlaty et al., 2014; Lan et al., 2020). Cell fate commitment requires efficient and timely control of the expression of pluripotency-associated factors. hm^5^C can promote a rapid response during differentiation processes (Cimmino et al., 2011; Kuehner et al., 2021). The RNA hm^5^C modification has been described as a guardian of the transcriptional landscape, able to regulate the balance between pluripotency and lineage-priming mRNAs and ensures ESC differentiation in a timely and orderly manner (Wu et al., 2018; Yang et al., 2020). TET-dependent hydroxymethylation of mRNA molecules can regulate RNA half-life and splicing. In mouse embryonic stem cells (mESCs) hm^5^C has been linked to the downregulation of certain mRNAs linked to pluripotency allowing ESC-to-EB (embryo body) differentiation (Lan et al., 2020). During somatic reprogramming to pluripotency, hm^5^C deposition drives site-specific demethylation of reprogramming enhancers and promoters resulting in the formation of iPSC (induced pluripotent stem cells) (Caldwell et al., 2021). TET triple knockout cells failed to undergo iPSC reprogramming, highlighting the importance of m^5^C hydroxylation during state transitions. Furthermore, a report on somatic reprogramming determined the contribution of hm^5^C deposition decoupled from the production of the oxidative derivatives f^5^C and ca^5^C using a hm^5^C-stalled TET enzyme. Interestingly, hm^5^C deposition alone is not sufficient for iPSC reprogramming, and f^5^C, ca^5^C and the TDG protein appeared to be crucial in this process, suggesting a different role of hm^5^C and the f^5^C and ca^5^C epigenetic signatures (Caldwell et al., 2021).

## 5-Hydroxymethylcytosine Modification in Cancer Onset

hm^5^C has been proposed to act as a novel diagnostic and prognostic marker in several human malignancies (Scourzic et al., 2015; An et al., 2017). Loss or inactivation of TET enzymes and deregulation of m^5^C demethylation are emerging as crucial determinants for cancer phenotypes. Modulation of the expression and activity of TET proteins can occur as a result of different mechanisms, such as m^5^C-mediated silencing of the TET loci or changes in the intracellular concentration of TET co-factors (i.e., α-Ketoglutarate, oxygen, iron, vitamin C) (Scourzic et al., 2015; Yue and Rao, 2020; Matuleviciute et al., 2021). Several studies have outlined a negative correlation between the loss of TET activity with increased levels of m^5^C and decreased levels of hm^5^C, and poor prognosis in cancers including lung, cervical, breast, glioblastoma, and hematopoietic (López-Moyado et al., 2019; Gao et al., 2021; Xu et al., 2021; Lopez-Bertoni et al., 2022; Xu et al., 2022). The deregulation of m^5^C and hm^5^C affects cell transcriptional programs and leads to cancer stem-like phenotypes. Moreover, aberrant deposition of hm^5^C has been also proposed as a contributing factor to chemotherapy resistance (Kharat and Sharan, 2020).

## 5-Hydroxymethylcytosine Modification in the DNA Damage Response

Of note, TET-mediated hm^5^C accumulation has also been described at DNA damage, suggesting a crucial role for hm^5^C in promoting DNA damage repair (Kafer et al., 2016). Possible mechanisms of action could be linked to the ability of hm^5^C to promote or maintain an open chromatin configuration. hm^5^C modification is able to control DNA accessibility through a DNA-end breathing motion that can decrease nucleosome affinities, facilitate RNA polymerase II elongation, and lower the thermodynamic stability of the DNA duplex (Mendonca et al., 2014; Li et al., 2022). Local accessibility of hm^5^C chromatin driven by hm^5^C, could serve as a platform for the recruitment of late-acting DNA damage repair factors. The hm^5^C modification has also been found to promote the formation of DNA:RNA hybrids (R-loops) *in vitro* and *in vivo* (Sabino et al., 2022; Shukla et al., 2022; Yang et al., 2022). Given that DNA:RNA hybrids are well-established triggers of DNA damage, hm^5^C modification has been proposed as novel player in genome instability. Several studies have documented a direct association between TET deficiency and increased level of DNA double strand breaks caused by the accumulation of R-loop structures. Ineffective m^5^C demethylation can further impair DNA damage repair through the retention of m^5^C readers on the R-loops and delayed R-loop resolution. Even if hm^5^C deposition in DNA damage responses is not fully understood, a direct correlation between the level of hm^5^C and fork stability has recently been described (Kharat et al., 2020). Upon DNA damage, ATM and ATR kinases can phosphorylate TET proteins and stimulate hm^5^C deposition close to the replication fork. The excessive presence of hm^5^C at replication forks triggers BER-mediated repair of hm^5^C, leading to the production of abasic sites which could sources of genome instability.

## Beyond 5-Methylcytosine: The Role of N6-Methyladenosine Modification

Dynamic and reversible chemical control of DNA and RNA also encompasses other methylated nucleobases. So far, several enzymatically methylated residues including N6-methyladenosine (m^6^A) (Zhu et al., 2020), N1-methyladenosine (m^1^A) (Xiong et al., 2018), N7-methylguanosine (m^7^G) (Enroth et al., 2019) and N4-methylcytosine (m^4^C) (Chen et al., 2020b) have been described in all major RNA types, while their presence in DNA is mainly restricted to m^6^A (Xu and Bochtler, 2020). For example, the existence of m^6^A in DNA has been suggested to be dependent on RNA catabolism rather than a specific m^6^A writer, arguing against the proposed role of m^6^A as a heritable epigenetic mark (Musheev et al., 2020). This evidence is also corroborated by the homogeneous distribution of m^6^A throughout the genome, implying an incorporation of m^6^A form the RNA nucleoside pool rather than from the direct action of a DNA methyltransferase. Although some reports have proposed a role for m^6^A in the epigenetic control of the heterochromatin formation, m^6^A-mediated regulation of chromatin dynamics appears to depend more on m^6^A methylated RNAs such as chromosome-associated regulatory RNAs (carRNAs) (Zhang et al., 2020b; Liu et al., 2020; Selmi and Lanzuolo, 2022) rather than a direct effect of m^6^A deposition in the genome. Nevertheless, m^6^A is a well-established RNA epitranscriptomic mark and its dynamical and reversible write and erase processes are mainly regulated by some members of the methyltransferase-like gene family (METTL3, METTL13 and METTL14) and FTO and ALKBH5 enzymes, respectively (Meyer and Jaffrey, 2014). The m^6^A writing process predominantly occurs in the nucleus, while reading and erasing events are reported in both the nucleus and cytoplasm. Distinctive subcellular localization of m^6^A erasers and readers confers the specific roles of the m^6^A residue, and thereby influences different pathways. m^6^A functions have been linked to several biological processes such as modulation of splicing by altering the structure of pre-mRNAs (Zhou et al., 2019), increased miRNA biogenesis by enhancing the recognition and processing of the microRNA microprocessor complex protein DGCR8 (Han et al., 2021), transcription termination by facilitating the co-transcriptional R-loops formation (Yang et al., 2019), regulation of DNA damage repair by the accumulation of DNA:RNA hybrids at DSB sites (Zhang et al., 2020c; Qu et al., 2021) development and pluripotency (Zhang et al., 2020b; Jin et al., 2021). Furthermore, it has been reported that m^6^A modification can influence mRNA export from the nucleus, determine transcripts turnover and stimulate translation initiation (Jiang et al., 2021). Perturbation of m^6^A deposition and elimination dynamics, due to upregulated or mutated enzymes, has been associated with tumor initiation and progression, metastasis, cell proliferation and self-renewal (He et al., 2019; Zhou et al., 2020). Moreover, opposite effects of m^6^A modification levels reported in some cancer settings (e.g., ovarian cancers), can be explained by the involvement of specific m^6^A readers in the stability of either oncogene or tumor-suppressor m^6^A-modified mRNAs (Chen et al., 2021b; Huang et al., 2022).

## Concluding Remarks

A growing body of evidence suggest that epigenetic and epitranscriptomic dynamics are profoundly interconnected. Methyltransferases (“writers”) and demethylases (“erasers”) functionally cooperate and compete to maintain the appropriate amount of m^5^C and its oxidized derivatives across both the genome and the transcriptome. Maintenance of effective, time regulated, and lineage-specific methylation-demethylation dynamics is needed for cellular homeostasis and responses to diverse stimuli. Mutations in epigenetic and epitranscriptomic modifiers can deregulate normal cellular differentiation and programmed growth control. As a consequence of altered patterns of hm^5^C and m^5^C, mainly characterised by global hypomethylation and focal hypermethylation, multiple stages of tumorigenesis including initiation, progression and metastasis, are promoted. Moreover, numerous reports correlate the loss of hm^5^C with poor prognosis. Restoration of proper methylation-demethylation dynamics in cells could be achieve with several approaches. Identification of druggable targets of both main players (DNMTs, TETs) and pivotal intermediates (miRNAs or lncRNAs) is crucial for the development of classical inhibitors or RNA-based drugs. A combination of epigenetic therapy and classical chemotherapy is a promising approach aiming at reducing tumour growth and self-renewal characteristics, while reducing chemoresistance (Hu et al., 2021b).

In addition, the intriguing possibility of precise and synchronized crosstalks between diverse epigenetic and epitranscriptomic marks (e.g., m^5^C and m^6^A) (Rengaraj et al., 2021) as versatile checkpoints in the maintenance of cellular homeostasis, may underpin a complex and comprehensive landscape of the cellular methylation game.

## References

[B1] Allis C. D. Jenuwein T. (2016). The Molecular Hallmarks of Epigenetic Control. Nat. Rev. Genet. 17, 487–500. 10.1038/nrg.2016.59 27346641

[B2] Amort T. Soulière M. F. Wille A. Jia X. Y. Fiegl H. Wörle H. (2013). Long Non-coding RNAs as Targets for Cytosine Methylation. RNA Biol. 10, 1003–1008. 10.4161/rna.24454 23595112 PMC4111728

[B3] Amort T. Rieder D. Wille A. Khokhlova-Cubberley D. Riml C. Trixl L. (2017). Distinct 5-methylcytosine Profiles in Poly(A) RNA from Mouse Embryonic Stem Cells and Brain. Genome Biol. 18, 1. 10.1186/s13059-016-1139-1 28077169 PMC5225599

[B4] An J. Rao A. Ko M. (2017). TET Family Dioxygenases and DNA Demethylation in Stem Cells and Cancers. Exp. Mol. Med. 49, e323. 10.1038/emm.2017.5 28450733 PMC6130217

[B5] Atala A. (2020). Re: 5-Methylcytosine Promotes Pathogenesis of Bladder Cancer through Stabilizing mRNAs. J. Urology 203, 884–885. 10.1097/ju.0000000000000781 32073983

[B6] Awah C. U. Winter J. Mazdoom C. M. Ogunwobi O. O. (2021). NSUN6, an RNA Methyltransferase of 5-mC Controls Glioblastoma Response to Temozolomide (TMZ) via NELFB and RPS6KB2 Interaction. Cancer Biol. Ther. 22, 587–597. 10.1080/15384047.2021.1990631 34705606 PMC8726740

[B7] Biswas S. Rao C. M. (2018). Epigenetic Tools (The Writers, The Readers and The Erasers) and Their Implications in Cancer Therapy. Eur. J. Pharmacol. 837, 8–24. 10.1016/j.ejphar.2018.08.021 30125562

[B8] Bohnsack K. E. Höbartner C. Bohnsack M. T. (2019). Eukaryotic 5-methylcytosine (m⁵C) RNA Methyltransferases: Mechanisms, Cellular Functions, and Links to Disease. Genes (Basel) 10, 102. 10.3390/genes10020102 PMC6409601 30704115

[B9] Bonasio R. Tu S. Reinberg D. (2010). Molecular Signals of Epigenetic States. Science 330, 612–616. 10.1126/science.1191078 21030644 PMC3772643

[B10] Boo S. H. Kim Y. K. (2020). The Emerging Role of RNA Modifications in the Regulation of mRNA Stability. Exp. Mol. Med. 52, 400–408. 10.1038/s12276-020-0407-z 32210357 PMC7156397

[B11] Bordin D. L. Lirussi L. Nilsen H. (2021). Cellular Response to Endogenous DNA Damage: DNA Base Modifications in Gene Expression Regulation. DNA Repair 99, 103051. 10.1016/j.dnarep.2021.103051 33540225

[B12] Buchmuller B. C. Kosel B. Summerer D. (2020). Complete Profiling of Methyl-CpG-Binding Domains for Combinations of Cytosine Modifications at CpG Dinucleotides Reveals Differential Read-Out in Normal and Rett-Associated States. Sci. Rep. 10, 4053. 10.1038/s41598-020-61030-1 32132616 PMC7055227

[B13] Caldwell B. A. Liu M. Y. Prasasya R. D. Wang T. DeNizio J. E. Leu N. A. (2021). Functionally Distinct Roles for TET-Oxidized 5-methylcytosine Bases in Somatic Reprogramming to Pluripotency. Mol. Cell 81, 859–869. 10.1016/j.molcel.2020.11.045 33352108 PMC7897302

[B14] Cervantes-Gracia K. Gramalla-Schmitz A. Weischedel J. Chahwan R. (2021). APOBECs Orchestrate Genomic and Epigenomic Editing across Health and Disease. Trends Genet. 37, 1028–1043. 10.1016/j.tig.2021.07.003 34353635

[B15] Chellamuthu A. Gray S. G. (2020). The RNA Methyltransferase NSUN2 and Its Potential Roles in Cancer. Cells 9, 1758. 10.3390/cells9081758 PMC7463552 32708015

[B16] Chen H. Shi Z. Guo J. Chang K.-j. Chen Q. Yao C.-H. (2020). The Human Mitochondrial 12S rRNA m4C Methyltransferase METTL15 Is Required for Mitochondrial Function. J. Biol. Chem. 295, 8505–8513. 10.1074/jbc.ra119.012127 32371392 PMC7307190

[B17] Chen H. Yang H. Zhu X. Yadav T. Ouyang J. Truesdell S. S. (2020). m5C Modification of mRNA Serves a DNA Damage Code to Promote Homologous recombinationC Modification of mRNA Serves a DNA Damage Code to Promote Homologous Recombination. Nat. Commun. 11, 2834. 10.1038/s41467-020-16722-7 32503981 PMC7275041

[B18] Chen X. Li A. Sun B.-F. Yang Y. Han Y.-N. Yuan X. (2019). 5-methylcytosine Promotes Pathogenesis of Bladder Cancer through Stabilizing mRNAs. Nat. Cell Biol. 21, 978–990. 10.1038/s41556-019-0361-y 31358969

[B19] Chen Y. S. Yang W. L. Zhao Y. L. Yang Y. G. (2021). Dynamic Transcriptomic M(5) C and its Regulatory Role in RNA Processing. Wiley Interdiscip. Rev. RNA. 12, e1639. 10.1002/wrna.1639 33438329

[B20] Chen Z. Zhong X. Xia M. Zhong J. (2021). The Roles and Mechanisms of the m6A Reader Protein YTHDF1 in Tumor Biology and Human Diseases. Mol. Ther. - Nucleic Acids 26, 1270–1279. 10.1016/j.omtn.2021.10.023 34853726 PMC8609105

[B21] Cheng J. X. Chen L. Li Y. Cloe A. Yue M. Wei J. (2018). RNA Cytosine Methylation and Methyltransferases Mediate Chromatin Organization and 5-azacytidine Response and Resistance in Leukaemia. Nat. Commun. 9, 1163. 10.1038/s41467-018-03513-4 29563491 PMC5862959

[B22] Choi I. Kim R. Lim H.-W. Kaestner K. H. Won K.-J. (2014). 5-hydroxymethylcytosine Represses the Activity of Enhancers in Embryonic Stem Cells: a New Epigenetic Signature for Gene Regulation. BMC Genomics 15, 670. 10.1186/1471-2164-15-670 25106691 PMC4133056

[B23] Cimmino L. Abdel-Wahab O. Levine R. L. Aifantis I. (2011). TET Family Proteins and Their Role in Stem Cell Differentiation and Transformation. Cell Stem Cell 9, 193–204. 10.1016/j.stem.2011.08.007 21885017 PMC3244690

[B24] Cui X.-L. Nie J. Ku J. Dougherty U. West-Szymanski D. C. Collin F. (2020). A Human Tissue Map of 5-hydroxymethylcytosines Exhibits Tissue Specificity through Gene and Enhancer Modulation. Nat. Commun. 11, 6161. 10.1038/s41467-020-20001-w 33268789 PMC7710742

[B25] Dai Z. Ramesh V. Locasale J. W. (2020). The Evolving Metabolic Landscape of Chromatin Biology and Epigenetics. Nat. Rev. Genet. 21, 737–753. 10.1038/s41576-020-0270-8 32908249 PMC8059378

[B26] Dawlaty M. M. Breiling A. Le T. Barrasa M. I. Raddatz G. Gao Q. (2014). Loss of Tet Enzymes Compromises Proper Differentiation of Embryonic Stem Cells. Dev. Cell 29, 102–111. 10.1016/j.devcel.2014.03.003 24735881 PMC4035811

[B27] Deem A. K. Li X. Tyler J. K. (2012). Epigenetic Regulation of Genomic Integrity. Chromosoma 121, 131–151. 10.1007/s00412-011-0358-1 22249206 PMC3982914

[B28] Delatte B. Wang F. Ngoc L. V. Collignon E. Bonvin E. Deplus R. (2016). Transcriptome-wide Distribution and Function of RNA Hydroxymethylcytosine. Science 351, 282–285. 10.1126/science.aac5253 26816380

[B29] DeNizio J. E. Liu M. Y. Leddin E. M. Cisneros G. A. Kohli R. M. (2019). Selectivity and Promiscuity in TET-Mediated Oxidation of 5-Methylcytosine in DNA and RNA. Biochemistry 58, 411–421. 10.1021/acs.biochem.8b00912 30387995 PMC6363868

[B30] Du Q. Luu P.-L. Stirzaker C. Clark S. J. (2015). Methyl-CpG-binding Domain Proteins: Readers of the Epigenome. Epigenomics 7, 1051–1073. 10.2217/epi.15.39 25927341

[B31] Enroth C. Poulsen L. D. Iversen S. Kirpekar F. Albrechtsen A. Vinther J. (2019). Detection of Internal N7-Methylguanosine (m7G) RNA Modifications by Mutational Profiling Sequencing. Nucleic Acids Res. 47, e126. 10.1093/nar/gkz736 31504776 PMC6847341

[B32] Esteller M. Pandolfi P. P. (2017). The Epitranscriptome of Noncoding RNAs in Cancer. Cancer Discov. 7, 359–368. 10.1158/2159-8290.cd-16-1292 28320778 PMC5997407

[B33] Esteve-Puig R. Bueno-Costa A. Esteller M. (2020). Writers, Readers and Erasers of RNA Modifications in Cancer. Cancer Lett. 474, 127–137. 10.1016/j.canlet.2020.01.021 31991154

[B34] Feng L.-y. Yan B.-b. Huang Y.-z. Li L. (2021). Abnormal Methylation Characteristics Predict Chemoresistance and Poor Prognosis in Advanced High-Grade Serous Ovarian Cancer. Clin. Epigenet 13, 141. 10.1186/s13148-021-01133-2 PMC8296752 34289901

[B35] Feng Y. Chen J.-J. Xie N.-B. Ding J.-H. You X.-J. Tao W.-B. (2021). Direct Decarboxylation of Ten-Eleven Translocation-Produced 5-carboxylcytosine in Mammalian Genomes Forms a New Mechanism for Active DNA Demethylation. Chem. Sci. 12, 11322–11329. 10.1039/d1sc02161c 34567494 PMC8409474

[B36] Flamand M. N. Meyer K. D. (2019). The Epitranscriptome and Synaptic Plasticity. Curr. Opin. Neurobiol. 59, 41–48. 10.1016/j.conb.2019.04.007 31108373 PMC6858947

[B37] Frye M. Harada B. T. Behm M. He C. (2018). RNA Modifications Modulate Gene Expression during Development. Science 361, 1346–1349. 10.1126/science.aau1646 30262497 PMC6436390

[B38] Fu L. Guerrero C. R. Zhong N. Amato N. J. Liu Y. Liu S. (2014). Tet-mediated Formation of 5-hydroxymethylcytosine in RNA. J. Am. Chem. Soc. 136, 11582–11585. 10.1021/ja505305z 25073028 PMC4140497

[B39] Gao J. Liu R. Feng D. Huang W. Huo M. Zhang J. (2021). Snail/PRMT5/NuRD Complex Contributes to DNA Hypermethylation in Cervical Cancer by TET1 Inhibition. Cell Death Differ. 28, 2818–2836. 10.1038/s41418-021-00786-z 33953349 PMC8408166

[B40] García-Vílchez R. Sevilla A. Blanco S. (2019). Post-transcriptional Regulation by Cytosine-5 Methylation of RNA. Biochimica Biophysica Acta (BBA) - Gene Regul. Mech. 1862, 240–252. 10.1016/j.bbagrm.2018.12.003 30593929

[B41] Genenncher B. Durdevic Z. Hanna K. Zinkl D. Mobin M. B. Senturk N. (2018). Mutations in Cytosine-5 tRNA Methyltransferases Impact Mobile Element Expression and Genome Stability at Specific DNA Repeats. Cell Rep. 22, 1861–1874. 10.1016/j.celrep.2018.01.061 29444437

[B42] George H. Ule J. Hussain S. (2017). Illustrating the Epitranscriptome at Nucleotide Resolution Using Methylation-iCLIP (miCLIP). Methods Mol. Biol. 1562, 91–106. 10.1007/978-1-4939-6807-7_7 28349456

[B43] Ginno P. A. Gaidatzis D. Feldmann A. Hoerner L. Imanci D. Burger L. (2020). A Genome-Scale Map of DNA Methylation Turnover Identifies Site-specific Dependencies of DNMT and TET Activity. Nat. Commun. 11, 2680. 10.1038/s41467-020-16354-x 32471981 PMC7260214

[B44] Gkatza N. A. Castro C. Harvey R. F. Heiss M. Popis M. C. Blanco S. (2019). Cytosine-5 RNA Methylation Links Protein Synthesis to Cell Metabolism. PLoS Biol. 17, e3000297. 10.1371/journal.pbio.3000297 31199786 PMC6594628

[B45] Guo G. Pan K. Fang S. Ye L. Tong X. Wang Z. (2021). Advances in mRNA 5-methylcytosine Modifications: Detection, Effectors, Biological Functions, and Clinical Relevance. Mol. Ther. - Nucleic Acids 26, 575–593. 10.1016/j.omtn.2021.08.020 34631286 PMC8479277

[B46] Han X. Guo J. Fan Z. P. (2021). Interactions between m6A Modification and miRNAs in Malignant Tumors. Cell Death Dis. 12, 598. 10.1038/s41419-021-03868-5 34108450 PMC8190295

[B47] He B. Zhang C. Zhang X. Fan Y. Zeng H. Liu J. e. (2021). Tissue-specific 5-hydroxymethylcytosine Landscape of the Human Genome. Nat. Commun. 12, 4249. 10.1038/s41467-021-24425-w 34253716 PMC8275684

[B48] He C. Bozler J. Janssen K. A. Wilusz J. E. Garcia B. A. Schorn A. J. (2021). TET2 Chemically Modifies tRNAs and Regulates tRNA Fragment Levels. Nat. Struct. Mol. Biol. 28, 62–70. 10.1038/s41594-020-00526-w 33230319 PMC7855721

[B49] He L. Li H. Wu A. Peng Y. Shu G. Yin G. (2019). Functions of N6-Methyladenosine and its Role in Cancer. Mol. Cancer 18, 176. 10.1186/s12943-019-1109-9 31801551 PMC6892141

[B50] He Y.-F. Li B.-Z. Li Z. Liu P. Wang Y. Tang Q. (2011). Tet-mediated Formation of 5-carboxylcytosine and its Excision by TDG in Mammalian DNA. Science 333, 1303–1307. 10.1126/science.1210944 21817016 PMC3462231

[B51] He Y. Shi Q. Zhang Y. Yuan X. Yu Z. (2020). Transcriptome-Wide 5-Methylcytosine Functional Profiling of Long Non-Coding RNA in Hepatocellular Carcinoma. Cmar 12, 6877–6885. 10.2147/cmar.s262450 PMC7414925 32801911

[B52] Hsu P. J. Shi H. He C. (2017). Epitranscriptomic Influences on Development and Disease. Genome Biol. 18, 197. 10.1186/s13059-017-1336-6 29061143 PMC5654102

[B53] Hu C. Liu X. Zeng Y. Liu J. Wu F. (2021). DNA Methyltransferase Inhibitors Combination Therapy for the Treatment of Solid Tumor: Mechanism and Clinical Application. Clin. Epigenet 13, 166. 10.1186/s13148-021-01154-x PMC8394595 34452630

[B54] Hu Y. Chen C. Tong X. Chen S. Hu X. Pan B. (2021). NSUN2 Modified by SUMO-2/3 Promotes Gastric Cancer Progression and Regulates mRNA m5C Methylation. Cell Death Dis. 12, 842. 10.1038/s41419-021-04127-3 34504059 PMC8429414

[B55] Huang T. Chen W. Liu J. Gu N. Zhang R. (2019). Genome-wide Identification of mRNA 5-methylcytosine in Mammals. Nat. Struct. Mol. Biol. 26, 380–388. 10.1038/s41594-019-0218-x 31061524

[B56] Huang W. Kong F. Li R. Chen X. Wang K. (2022). Emerging Roles of m6A RNA Methylation Regulators in Gynecological Cancer. Front. Oncol. 12, 827956. 10.3389/fonc.2022.827956 35155260 PMC8831694

[B57] Huang Z.-X. Li J. Xiong Q.-P. Li H. Wang E.-D. Liu R.-J. (2021). Position 34 of tRNA Is a Discriminative Element for m5C38 Modification by Human DNMT2. Nucleic Acids Res. 49, 13045–13061. 10.1093/nar/gkab1148 34871455 PMC8682788

[B58] Huang Z. Pan J. Wang H. Du X. Xu Y. Wang Z. (2021). Prognostic Significance and Tumor Immune Microenvironment Heterogenicity of m5C RNA Methylation Regulators in Triple-Negative Breast Cancer. Front. Cell Dev. Biol. 9, 657547. 10.3389/fcell.2021.657547 33928086 PMC8076743

[B59] Ibrahim J. Op de Beeck K. Fransen E. Peeters M. Van Camp G. (2022). Genome-wide DNA Methylation Profiling and Identification of Potential Pan-Cancer and Tumor-specific Biomarkers. Mol. Oncol. 10.1002/1878-0261.13176 PMC9208075 34978357

[B60] Ito S. Shen L. Dai Q. Wu S. C. Collins L. B. Swenberg J. A. (2011). Tet Proteins Can Convert 5-methylcytosine to 5-formylcytosine and 5-carboxylcytosine. Science 333, 1300–1303. 10.1126/science.1210597 21778364 PMC3495246

[B61] Jiang Q. Crews L. A. Holm F. Jamieson C. H. M. (2017). RNA Editing-dependent Epitranscriptome Diversity in Cancer Stem Cells. Nat. Rev. Cancer 17, 381–392. 10.1038/nrc.2017.23 28416802 PMC5665169

[B62] Jiang X. L. Liu B. Y. Nie Z. Duan L. C. Xiong Q. X. Jin Z. X. (2021). The Role of m6A Modification in the Biological Functions and Diseases. Signal Transduct. Tar 6, 74. 10.1038/s41392-020-00450-x PMC7897327 33611339

[B63] Jimeno S. Balestra F. R. Huertas P. (2021). The Emerging Role of RNA Modifications in DNA Double-Strand Break Repair. Front. Mol. Biosci. 8, 664872. 10.3389/fmolb.2021.664872 33996910 PMC8116738

[B64] Jin K. X. Zuo R. J. Anastassiadis K. Klungland A. Marr C. Filipczyk A. (2021). N6-methyladenosine (m(6)A) Depletion Regulates Pluripotency Exit by Activating Signaling Pathways in Embryonic Stem Cells. P Natl. Acad. Sci. U. S. A. 118, e2105192118. 10.1073/pnas.2105192118 PMC8713808 34921114

[B65] John R. M. Rougeulle C. (2018). Developmental Epigenetics: Phenotype and the Flexible Epigenome. Front. Cell Dev. Biol. 6, 130. 10.3389/fcell.2018.00130 30364270 PMC6193064

[B66] Kafer G. R. Li X. Horii T. Suetake I. Tajima S. Hatada I. (2016). 5-Hydroxymethylcytosine Marks Sites of DNA Damage and Promotes Genome Stability. Cell Rep. 14, 1283–1292. 10.1016/j.celrep.2016.01.035 26854228

[B67] Kan R. L. Chen J. Sallam T. (2022). Crosstalk between Epitranscriptomic and Epigenetic Mechanisms in Gene Regulation. Trends Genet. 38, 182–193. 10.1016/j.tig.2021.06.014 34294427 PMC9093201

[B68] Ketley R. F. Gullerova M. (2020). Jack of All Trades? The Versatility of RNA in DNA Double-Strand Break Repair. Essays Biochem. 64, 721–735. 10.1042/ebc20200008 32618336 PMC7592198

[B69] Kharat S. S. Ding X. Swaminathan D. Suresh A. Singh M. Sengodan S. K. (2020). Degradation of 5hmC-Marked Stalled Replication Forks by APE1 Causes Genomic Instability. Sci. Signal 13, eaba8091. 10.1126/scisignal.aba8091 32817374 PMC7575062

[B70] Kharat S. S. Sharan S. K. (2020). Exploring Role of 5hmC as Potential Marker of Chemoresistance. Mol. Cell. Oncol. 7, 1827904. 10.1080/23723556.2020.1827904 33235920 PMC7671001

[B71] Khoddami V. Cairns B. R. (2013). Identification of Direct Targets and Modified Bases of RNA Cytosine Methyltransferases. Nat. Biotechnol. 31, 458–464. 10.1038/nbt.2566 23604283 PMC3791587

[B72] Kim S. Kaang B.-K. (2017). Epigenetic Regulation and Chromatin Remodeling in Learning and Memory. Exp. Mol. Med. 49, e281. 10.1038/emm.2016.140 28082740 PMC5291841

[B73] Kuehner J. N. Chen J. Bruggeman E. C. Wang F. Li Y. Xu C. (2021). 5-hydroxymethylcytosine Is Dynamically Regulated during Forebrain Organoid Development and Aberrantly Altered in Alzheimer's Disease. Cell Rep. 35, 109042. 10.1016/j.celrep.2021.109042 33910000 PMC8106871

[B74] Kumar S. Chinnusamy V. Mohapatra T. (2018). Epigenetics of Modified DNA Bases: 5-Methylcytosine and Beyond. Front. Genet. 9, 640. 10.3389/fgene.2018.00640 30619465 PMC6305559

[B75] Kumar S. Mohapatra T. (2021). Deciphering Epitranscriptome: Modification of mRNA Bases Provides a New Perspective for Post-transcriptional Regulation of Gene Expression. Front. Cell Dev. Biol. 9, 628415. 10.3389/fcell.2021.628415 33816473 PMC8010680

[B76] Lan J. Rajan N. Bizet M. Penning A. Singh N. K. Guallar D. (2020). Functional Role of Tet-Mediated RNA Hydroxymethylcytosine in Mouse ES Cells and during Differentiation. Nat. Commun. 11, 4956. 10.1038/s41467-020-18729-6 33009383 PMC7532169

[B77] Lee M. P. (2019). Understanding Cancer Through the Lens of Epigenetic Inheritance, Allele-Specific Gene Expression, and High-Throughput Technology. Front. Oncol. 9, 794. 10.3389/fonc.2019.00794 31497535 PMC6712412

[B78] Li H. Jiang H. Huang Z. Chen Z. Chen N. (2021). Prognostic Value of an m5C RNA Methylation Regulator-Related Signature for Clear Cell Renal Cell Carcinoma. Cmar 13, 6673–6687. 10.2147/cmar.s323072 PMC8404088 34471382

[B79] Li H. Zhu D. Wu J. Ma Y. Cai C. Chen Y. (2021). New Substrates and Determinants for tRNA Recognition of RNA Methyltransferase DNMT2/TRDMT1. RNA Biol. 18, 2531–2545. 10.1080/15476286.2021.1930756 34110975 PMC8632113

[B80] Li S. Peng Y. Landsman D. Panchenko A. R. (2022). DNA Methylation Cues in Nucleosome Geometry, Stability and Unwrapping. Nucleic Acids Res. 50, 1864–1874. 10.1093/nar/gkac097 35166834 PMC8881801

[B81] Lian H. Wang Q.-H. Zhu C.-B. Ma J. Jin W.-L. (2018). Deciphering the Epitranscriptome in Cancer. Trends Cancer 4, 207–221. 10.1016/j.trecan.2018.01.006 29506671

[B82] Liu J. Cui X. Jiang J. Cao D. He Y. Wang H. (2017). Uncoordinated Expression of DNA Methylation-Related Enzymes in Human Cancer. Epigenetics Chromatin. 10, 61. 10.1186/s13072-017-0170-0 29233176 PMC5727647

[B83] Liu J. Dou X. Chen C. Chen C. Liu C. Xu M. M. (2020). N 6 -methyladenosine of Chromosome-Associated Regulatory RNA Regulates Chromatin State and Transcription. Science 367, 580–586. 10.1126/science.aay6018 31949099 PMC7213019

[B84] Liu Y. Santi D. V. (2000). m5C RNA and M5C DNA Methyl Transferases Use Different Cysteine Residues as Catalysts. Proc. Natl. Acad. Sci. U.S.A. 97, 8263–8265. 10.1073/pnas.97.15.8263 10899996 PMC26935

[B85] Lobo J. Barros-Silva D. Henrique R. Jerónimo C. (2018). The Emerging Role of Epitranscriptomics in Cancer: Focus on Urological Tumors. Genes (Basel) 9, 552. 10.3390/genes9110552 PMC6265908 30428628

[B86] Locke W. J. Guanzon D. Ma C. Liew Y. J. Duesing K. R. Fung K. Y. C. (2019). DNA Methylation Cancer Biomarkers: Translation to the Clinic. Front. Genet. 10, 1150. 10.3389/fgene.2019.01150 31803237 PMC6870840

[B87] Lopez J. Anazco-Guenkova A. M. Monteagudo-Garcia O. Blanco S. (2022). Epigenetic and Epitranscriptomic Control in Prostate Cancer. Genes (Basel) 13, 378. 10.3390/genes13020378 35205419 PMC8872343

[B88] Lopez-Bertoni H. Johnson A. Rui Y. Lal B. Sall S. Malloy M. (2022). Sox2 Induces Glioblastoma Cell Stemness and Tumor Propagation by Repressing TET2 and Deregulating 5hmC and 5mC DNA Modifications. Sig Transduct. Target Ther. 7, 37. 10.1038/s41392-021-00857-0 PMC8826438 35136034

[B89] López-Moyado I. F. Tsagaratou A. Yuita H. Seo H. Delatte B. Heinz S. (2019). Paradoxical Association of TET Loss of Function with Genome-wide DNA Hypomethylation. Proc. Natl. Acad. Sci. U.S.A. 116, 16933–16942. 10.1073/pnas.1903059116 31371502 PMC6708373

[B90] Lu Y. Chan Y.-T. Tan H.-Y. Li S. Wang N. Feng Y. (2020). Epigenetic Regulation in Human Cancer: the Potential Role of Epi-Drug in Cancer Therapy. Mol. Cancer. 19, 79. 10.1186/s12943-020-01197-3 32340605 PMC7184703

[B91] Lyko F. (2018). The DNA Methyltransferase Family: a Versatile Toolkit for Epigenetic Regulation. Nat. Rev. Genet. 19, 81–92. 10.1038/nrg.2017.80 29033456

[B92] Ma J. Song B. Wei Z. Huang D. Zhang Y. Su J. (2022). m5C-Atlas: a Comprehensive Database for Decoding and Annotating the 5-methylcytosine (m5C) Epitranscriptome. Nucleic Acids Res. 50, D196–D203. 10.1093/nar/gkab1075 34986603 PMC8728298

[B93] Matuleviciute R. Cunha P. P. Johnson R. S. Foskolou I. P. (2021). Oxygen Regulation of TET Enzymes. FEBS J. 288, 7143–7161. 10.1111/febs.15695 33410283

[B94] McCarrey J. R. Lehle J. D. Raju S. S. Wang Y. Nilsson E. E. Skinner M. K. (2016). Tertiary Epimutations - A Novel Aspect of Epigenetic Transgenerational Inheritance Promoting Genome Instability. PLoS One. 11, e0168038. 10.1371/journal.pone.0168038 27992467 PMC5167269

[B95] Mendonca A. Chang E. H. Liu W. Yuan C. (2014). Hydroxymethylation of DNA Influences Nucleosomal Conformation and Stability *In Vitro* . Biochimica Biophysica Acta (BBA) - Gene Regul. Mech. 1839, 1323–1329. 10.1016/j.bbagrm.2014.09.014 25263161

[B96] Meyer K. D. Jaffrey S. R. (2014). The Dynamic Epitranscriptome: N6-Methyladenosine and Gene Expression Control. Nat. Rev. Mol. Cell Biol. 15, 313–326. 10.1038/nrm3785 24713629 PMC4393108

[B97] Miano V. Codino A. Pandolfini L. Barbieri I. (2021). The Non-coding Epitranscriptome in Cancer. Brief. Funct. Genomics 20, 94–105. 10.1093/bfgp/elab003 33564819 PMC8479548

[B98] Misiak B. Ricceri L. Sąsiadek M. M. (2019). Transposable Elements and Their Epigenetic Regulation in Mental Disorders: Current Evidence in the Field. Front. Genet. 10, 580. 10.3389/fgene.2019.00580 31293617 PMC6603224

[B99] Moore L. D. Le T. Fan G. (2013). DNA Methylation and its Basic Function. Neuropsychopharmacol 38, 23–38. 10.1038/npp.2012.112 PMC3521964 22781841

[B100] Musheev M. U. Baumgärtner A. Krebs L. Niehrs C. (2020). The Origin of Genomic N6-Methyl-Deoxyadenosine in Mammalian Cells. Nat. Chem. Biol. 16, 630–634. 10.1038/s41589-020-0504-2 32203414

[B101] Nishiyama A. Nakanishi M. (2021). Navigating the DNA Methylation Landscape of Cancer. Trends Genet. 37, 1012–1027. 10.1016/j.tig.2021.05.002 34120771

[B102] Nombela P. Miguel-López B. Blanco S. (2021). The Role of m6A, m5C and Ψ RNA Modifications in Cancer: Novel Therapeutic Opportunities. Mol. Cancer 20, 18. 10.1186/s12943-020-01263-w 33461542 PMC7812662

[B103] Norouzitallab P. Baruah K. Vanrompay D. Bossier P. (2019). Can Epigenetics Translate Environmental Cues into Phenotypes? Sci. Total Environ. 647, 1281–1293. 10.1016/j.scitotenv.2018.08.063 30180336

[B104] Oey H. Whitelaw E. (2014). On the Meaning of the Word 'epimutation'. Trends Genet. 30, 519–520. 10.1016/j.tig.2014.08.005 25301328

[B105] Okamoto M. Hirata S. Sato S. Koga S. Fujii M. Qi G. (2012). Frequent Increased Gene Copy Number and High Protein Expression of tRNA (Cytosine-5-)-methyltransferase (NSUN2) in Human Cancers. DNA Cell Biol. 31, 660–671. 10.1089/dna.2011.1446 22136356

[B106] Pérez R. F. Tejedor J. R. Bayón G. F. Fernández A. F. Fraga M. F. (2018). Distinct Chromatin Signatures of DNA Hypomethylation in Aging and Cancer. Aging Cell 17, e12744. 10.1111/acel.12744 29504244 PMC5946083

[B107] Popis M. C. Blanco S. Frye M. (2016). Posttranscriptional Methylation of Transfer and Ribosomal RNA in Stress Response Pathways, Cell Differentiation, and Cancer. Curr. Opin. Oncol. 28, 65–71. 10.1097/cco.0000000000000252 26599292 PMC4805175

[B108] Porcellini E. Laprovitera N. Riefolo M. Ravaioli M. Garajova I. Ferracin M. (2018). Epigenetic and Epitranscriptomic Changes in Colorectal Cancer: Diagnostic, Prognostic, and Treatment Implications. Cancer Lett. 419, 84–95. 10.1016/j.canlet.2018.01.049 29360561

[B109] Qu F. Tsegay P. S. Liu Y. (2021). N-6-Methyladenosine, DNA Repair, and Genome Stability. Front. Mol. Biosci. 8, 645823. 10.3389/fmolb.2021.645823 33898522 PMC8062805

[B110] Ranjan N. Leidel S. A. (2019). The Epitranscriptome in Translation Regulation: mRNA and tRNA Modifications as the Two Sides of the Same Coin? FEBS Lett. 593, 1483–1493. 10.1002/1873-3468.13491 31206634

[B111] Rengaraj P. Obrdlík A. Vukić D. Varadarajan N. M. Keegan L. P. Vaňáčová Š. (2021). Interplays of Different Types of Epitranscriptomic mRNA Modifications. Rna Biol. 18, 19–30. 10.1080/15476286.2021.1969113 34424827 PMC8677042

[B112] Romero-Garcia S. Prado-Garcia H. Carlos-Reyes A. (2020). Role of DNA Methylation in the Resistance to Therapy in Solid Tumors. Front. Oncol. 10, 1152. 10.3389/fonc.2020.01152 32850327 PMC7426728

[B113] Ruiz de la Cruz M. de la Cruz Montoya A. H. Rojas Jimenez E. A. Martinez Gregorio H. Diaz Velasquez C. E. Paredes de la Vega J. (2021). Cis-Acting Factors Causing Secondary Epimutations: Impact on the Risk for Cancer and Other Diseases. Cancers (Basel). 13, 4807. 10.3390/cancers13194807 34638292 PMC8508567

[B114] Sabino J. C. de Almeida M. R. Abreu P. L. Ferreira A. M. Caldas P. Domingues M. M. (2022). Epigenetic Reprogramming by TET Enzymes Impacts Co-transcriptional R-Loops. Elife 11, e69476. 10.7554/elife.69476 35191837 PMC8896830

[B115] Sadakierska-Chudy A. Kostrzewa R. M. Filip M. (2015). A Comprehensive View of the Epigenetic Landscape Part I: DNA Methylation, Passive and Active DNA Demethylation Pathways and Histone Variants. Neurotox. Res. 27, 84–97. 10.1007/s12640-014-9497-5 25362550 PMC4286137

[B116] Sajini A. A. Choudhury N. R. Wagner R. E. Bornelöv S. Selmi T. Spanos C. (2019). Loss of 5-methylcytosine Alters the Biogenesis of Vault-Derived Small RNAs to Coordinate Epidermal Differentiation. Nat. Commun. 10, 2550. 10.1038/s41467-019-10020-7 31186410 PMC6560067

[B117] Schaefer M. Kapoor U. Jantsch M. F. (2017). Understanding RNA Modifications: the Promises and Technological Bottlenecks of the 'epitranscriptome'. Open Biol. 7, 170077. 10.1098/rsob.170077 28566301 PMC5451548

[B118] Schaefer M. R. (2021). The Regulation of RNA Modification Systems: The Next Frontier in Epitranscriptomics? Genes (Basel) 12, 345. 10.3390/genes12030345 33652758 PMC7996938

[B119] Schosserer M. Minois N. Angerer T. B. Amring M. Dellago H. Harreither E. (2015). Methylation of Ribosomal RNA by NSUN5 Is a Conserved Mechanism Modulating Organismal Lifespan. Nat. Commun. 6, 6158. 10.1038/ncomms7158 25635753 PMC4317494

[B120] Schumann U. Zhang H.-N. Sibbritt T. Pan A. Horvath A. Gross S. (2020). Multiple Links between 5-methylcytosine Content of mRNA and Translation. BMC Biol. 18, 40. 10.1186/s12915-020-00769-5 32293435 PMC7158060

[B121] Scourzic L. Mouly E. Bernard O. A. (2015). TET Proteins and the Control of Cytosine Demethylation in Cancer. Genome Med. 7, 9. 10.1186/s13073-015-0134-6 25632305 PMC4308928

[B122] Selmi T. Lanzuolo C. (2022). Driving Chromatin Organisation through N6-Methyladenosine Modification of RNA: What Do We Know and What Lies Ahead? Genes-Basel 13, 340. 10.3390/genes13020340 35205384 PMC8871937

[B123] Seo K. W. Kleiner R. E. (2021). Mechanisms of Epitranscriptomic Gene Regulation. Biopolymers 112, e23403. 10.1002/bip.23403 33001446 PMC7855325

[B124] Shukla V. Samaniego-Castruita D. Dong Z. González-Avalos E. Yan Q. Sarma K. (2022). TET Deficiency Perturbs Mature B Cell Homeostasis and Promotes Oncogenesis Associated with Accumulation of G-Quadruplex and R-Loop Structures. Nat. Immunol. 23, 99–108. 10.1038/s41590-021-01087-w 34937926 PMC8772520

[B125] Song C.-X. He C. (2013). Potential Functional Roles of DNA Demethylation Intermediates. Trends Biochem. Sci. 38, 480–484. 10.1016/j.tibs.2013.07.003 23932479 PMC4013277

[B126] Song H. Zhang J. Liu B. Xu J. Cai B. Yang H. (2022). Biological Roles of RNA m5C Modification and its Implications in Cancer Immunotherapy. Biomark. Res. 10, 15. 10.1186/s40364-022-00362-8 35365216 PMC8973801

[B127] Su J. Wu G. Ye Y. Zhang J. Zeng L. Huang X. (2021). NSUN2-mediated RNA 5-methylcytosine Promotes Esophageal Squamous Cell Carcinoma Progression via LIN28B-dependent GRB2 mRNA Stabilization. Oncogene 40, 5814–5828. 10.1038/s41388-021-01978-0 34345012 PMC8484015

[B128] Sun R. Du C. Li J. Zhou Y. Xiong W. Xiang J. (2021). Systematic Investigation of DNA Methylation Associated With Platinum Chemotherapy Resistance Across 13 Cancer Types. Front. Pharmacol. 12, 616529. 10.3389/fphar.2021.616529 33995018 PMC8117351

[B129] Sun X. Huang X. Lu X. Wang N. Wu D. Yuan M. (2021). The Expression and Clinical Significance of the tRNA Aspartic Acid Methyltransferase 1 Protein in Gastric Cancer. Int. J. Clin. Oncol. 26, 2229–2236. 10.1007/s10147-021-02019-2 34689291

[B130] Sun Z. Xue S. Xu H. Hu X. Chen S. Yang Z. (2019). Effects of NSUN2 Deficiency on the mRNA 5-methylcytosine Modification and Gene Expression Profile in HEK293 Cells. Epigenomics 11, 439–453. 10.2217/epi-2018-0169 30526041

[B131] Sun Z. Xue S. Zhang M. Xu H. Hu X. Chen S. (2020). Aberrant NSUN2-Mediated m5C Modification of H19 lncRNA Is Associated with Poor Differentiation of Hepatocellular Carcinoma. Oncogene 39, 6906–6919. 10.1038/s41388-020-01475-w 32978516 PMC7644462

[B132] Trixl L. Lusser A. (2019). The Dynamic RNA Modification 5-methylcytosine and its Emerging Role as an Epitranscriptomic Mark. Wiley Interdiscip. Rev. RNA 10, e1510. 10.1002/wrna.1510 30311405 PMC6492194

[B133] Van Haute L. Lee S. Y. McCann B. J. Powell C. A. Bansal D. Vasiliauskaitė L. (2019). NSUN2 Introduces 5-methylcytosines in Mammalian Mitochondrial tRNAs. Nucleic Acids Res. 47, 8720–8733. 10.1093/nar/gkz559 31276587 PMC6822013

[B134] Vincenzetti L. Leoni C. Chirichella M. Kwee I. Monticelli S. (2019). The Contribution of Active and Passive Mechanisms of 5mC and 5hmC Removal in Human T Lymphocytes Is Differentiation‐ and Activation‐dependent. Eur. J. Immunol. 49, 611–625. 10.1002/eji.201847967 30698829

[B135] Wang L. Zhang J. Su Y. Maimaitiyiming Y. Yang S. Shen Z. (2022). Distinct Roles of m5C RNA Methyltransferase NSUN2 in Major Gynecologic Cancers. Front. Oncol. 12, 786266. 10.3389/fonc.2022.786266 35280737 PMC8916577

[B136] Weber A. R. Krawczyk C. Robertson A. B. Kuśnierczyk A. Vågbø C. B. Schuermann D. (2016). Biochemical Reconstitution of TET1-TDG-BER-dependent Active DNA Demethylation Reveals a Highly Coordinated Mechanism. Nat. Commun. 7, 10806. 10.1038/ncomms10806 26932196 PMC4778062

[B137] Wilkinson E. Cui Y. H. He Y. Y. (2021). Context-Dependent Roles of RNA Modifications in Stress Responses and Diseases. Int. J. Mol. Sci. 22, 1949. 10.3390/ijms22041949 33669361 PMC7920320

[B138] Willbanks A. Wood S. Cheng J. X. (2021). RNA Epigenetics: Fine-Tuning Chromatin Plasticity and Transcriptional Regulation, and the Implications in Human Diseases. Genes (Basel) 12, 627. 10.3390/genes12050627 33922187 PMC8145807

[B139] Wu H. Zhang Y. (2011). Mechanisms and Functions of Tet Protein-Mediated 5-methylcytosine Oxidation. Genes Dev. 25, 2436–2452. 10.1101/gad.179184.111 22156206 PMC3243055

[B140] Wu H. Zhang Y. (2014). Reversing DNA Methylation: Mechanisms, Genomics, and Biological Functions. Cell 156, 45–68. 10.1016/j.cell.2013.12.019 24439369 PMC3938284

[B141] Wu X. Li G. Xie R. (2018). Decoding the Role of TET Family Dioxygenases in Lineage Specification. Epigenetics Chromatin 11, 58. 10.1186/s13072-018-0228-7 30290828 PMC6172806

[B142] Xiang S. Ma Y. Shen J. Zhao Y. Wu X. Li M. (2020). m5C RNA Methylation Primarily Affects the ErbB and PI3K-Akt Signaling Pathways in Gastrointestinal CancerC RNA Methylation Primarily Affects the ErbB and PI3K-Akt Signaling Pathways in Gastrointestinal Cancer. Front. Mol. Biosci. 7, 599340. 10.3389/fmolb.2020.599340 33365328 PMC7750483

[B143] Xie S. Chen W. Chen K. Chang Y. Yang F. Lin A. (2020). Emerging Roles of RNA Methylation in Gastrointestinal Cancers. Cancer Cell Int. 20, 585. 10.1186/s12935-020-01679-w 33372610 PMC7720447

[B144] Xiong X. Li X. Yi C. (2018). N1-methyladenosine Methylome in Messenger RNA and Non-coding RNA. Curr. Opin. Chem. Biol. 45, 179–186. 10.1016/j.cbpa.2018.06.017 30007213

[B145] Xu B. Wang H. Tan L. (2021). Dysregulated TET Family Genes and Aberrant 5mC Oxidation in Breast Cancer: Causes and Consequences. Cancers (Basel) 13, 6039. 10.3390/cancers13236039 34885145 PMC8657367

[B146] Xu G.-L. Bochtler M. (2020). Reversal of Nucleobase Methylation by Dioxygenases. Nat. Chem. Biol. 16, 1160–1169. 10.1038/s41589-020-00675-5 33067602

[B147] Xu Q. Wang C. Zhou J. X. Xu Z. M. Gao J. Sui P. (2022). Loss of TET Reprograms Wnt Signaling through Impaired Demethylation to Promote Lung Cancer Development. Proc. Natl. Acad. Sci. U. S. A. 119, e2107599119. 10.1073/pnas.2107599119 35110400 PMC8832965

[B148] Xue C. Zhao Y. Li L. (2020). Advances in RNA Cytosine-5 Methylation: Detection, Regulatory Mechanisms, Biological Functions and Links to Cancer. Biomark. Res. 8, 43. 10.1186/s40364-020-00225-0 32944246 PMC7490858

[B149] Xue M. Shi Q. Zheng L. Li Q. Yang L. Zhang Y. (2020). Gene Signatures of m5C Regulators May Predict Prognoses of Patients with Head and Neck Squamous Cell Carcinoma. Am. J. Transl. Res. 12, 6841–6852. 33194076 PMC7653571

[B150] Yang H. Wang Y. Xiang Y. Yadav T. Ouyang J. Phoon L. (2022). FMRP Promotes Transcription-Coupled Homologous Recombination via Facilitating TET1-Mediated m5C RNA Modification Demethylation. Proc. Natl. Acad. Sci. U. S. A. 119, e2116251119. 10.1073/pnas.2116251119 35290126 PMC8944906

[B151] Yang J. Bashkenova N. Zang R. Huang X. Wang J. (2020). The Roles of TET Family Proteins in Development and Stem Cells. Development 147, dev183129. 10.1242/dev.183129 31941705 PMC6983710

[B152] Yang X. Liu Q.-L. Xu W. Zhang Y.-C. Yang Y. Ju L.-F. (2019). m6A Promotes R-Loop Formation to Facilitate Transcription terminationA Promotes R-Loop Formation to Facilitate Transcription Termination. Cell Res. 29, 1035–1038. 10.1038/s41422-019-0235-7 31606733 PMC6951339

[B153] Yi J. Gao R. Chen Y. Yang Z. Han P. Zhang H. (2017). Overexpression of NSUN2 by DNA Hypomethylation Is Associated with Metastatic Progression in Human Breast Cancer. Oncotarget 8, 20751–20765. 10.18632/oncotarget.10612 27447970 PMC5400542

[B154] Yuan F. Bi Y. Siejka-Zielinska P. Zhou Y.-L. Zhang X.-X. Song C.-X. (2019). Bisulfite-free and Base-Resolution Analysis of 5-methylcytidine and 5-hydroxymethylcytidine in RNA with Peroxotungstate. Chem. Commun. 55, 2328–2331. 10.1039/c9cc00274j PMC6984333 30723849

[B155] Yue X. Rao A. (2020). TET Family Dioxygenases and the TET Activator Vitamin C in Immune Responses and Cancer. Blood 136, 1394–1401. 10.1182/blood.2019004158 32730592 PMC7498365

[B156] Zeng Y. Wang S. Gao S. Soares F. Ahmed M. Guo H. (2018). Refined RIP-Seq Protocol for Epitranscriptome Analysis with Low Input Materials. PLoS Biol. 16, e2006092. 10.1371/journal.pbio.2006092 30212448 PMC6136692

[B157] Zhang C. Chen L. Peng D. Jiang A. He Y. Zeng Y. (2020). METTL3 and N6-Methyladenosine Promote Homologous Recombination-Mediated Repair of DSBs by Modulating DNA-RNA Hybrid Accumulation. Mol. Cell 79, 425–442. 10.1016/j.molcel.2020.06.017 32615088

[B158] Zhang M. Zhai Y. Zhang S. Dai X. Li Z. (2020). Roles of N6-Methyladenosine (m6A) in Stem Cell Fate Decisions and Early Embryonic Development in Mammals. Front. Cell Dev. Biol. 8, 782. 10.3389/fcell.2020.00782 32850871 PMC7431753

[B159] Zhang Q. Liu F. Chen W. Miao H. Liang H. Liao Z. (2021). The Role of RNA m5C Modification in Cancer Metastasis. Int. J. Biol. Sci. 17, 3369–3380. 10.7150/ijbs.61439 34512153 PMC8416729

[B160] Zhang Q. Zheng Q. Yu X. He Y. Guo W. (2020). Overview of Distinct 5-methylcytosine Profiles of Messenger RNA in Human Hepatocellular Carcinoma and Paired Adjacent Non-tumor Tissues. J. Transl. Med. 18, 245. 10.1186/s12967-020-02417-6 32571340 PMC7310161

[B161] Zhang X. Gan Y. Zou G. Guan J. Zhou S. (2019). Genome-wide Analysis of Epigenetic Dynamics across Human Developmental Stages and Tissues. BMC Genomics 20, 221. 10.1186/s12864-019-5472-0 30967107 PMC6457072

[B162] Zhao L.-Y. Song J. Liu Y. Song C.-X. Yi C. (2020). Mapping the Epigenetic Modifications of DNA and RNA. Protein Cell 11, 792–808. 10.1007/s13238-020-00733-7 32440736 PMC7647981

[B163] Zhao L. Ma S. Wang L. Wang Y. Feng X. Liang D. (2021). A Polygenic Methylation Prediction Model Associated with Response to Chemotherapy in Epithelial Ovarian Cancer. Mol. Ther. - Oncolytics 20, 545–555. 10.1016/j.omto.2021.02.012 33738340 PMC7943968

[B164] Zhou K. I. Shi H. Lyu R. Wylder A. C. Matuszek Ż. Pan J. N. (2019). Regulation of Co-transcriptional Pre-mRNA Splicing by m6A through the Low-Complexity Protein hnRNPG. Mol. Cell 76, 70–81. e9. 10.1016/j.molcel.2019.07.005 31445886 PMC6778029

[B165] Zhou Z. Lv J. Yu H. Han J. Yang X. Feng D. (2020). Mechanism of RNA Modification N6-Methyladenosine in Human Cancer. Mol. Cancer 19, 104. 10.1186/s12943-020-01216-3 32513173 PMC7278081

[B166] Zhu X. Wang X. Yan W. Yang H. Xiang Y. Lv F. (2021). Ubiquitination-mediated Degradation of TRDMT1 Regulates Homologous Recombination and Therapeutic Response. Nar. Cancer 3, zcab010. 10.1093/narcan/zcab010 33778494 PMC7984809

[B167] Zhu Z.-M. Huo F.-C. Pei D.-S. (2020). Function and Evolution of RNA N6-Methyladenosine Modification. Int. J. Biol. Sci. 16, 1929–1940. 10.7150/ijbs.45231 32398960 PMC7211178

