# Supplementary figures and images for: The Methylation Game: Epigenetic and Epitranscriptomic Dynamics of 5-Methylcytosine
Source: Front Cell Dev Biol. 2022 Jun 3;10:915685. doi: 10.3389/fcell.2022.915685 (PMC9204050; doi:10.3389/fcell.2022.915685)

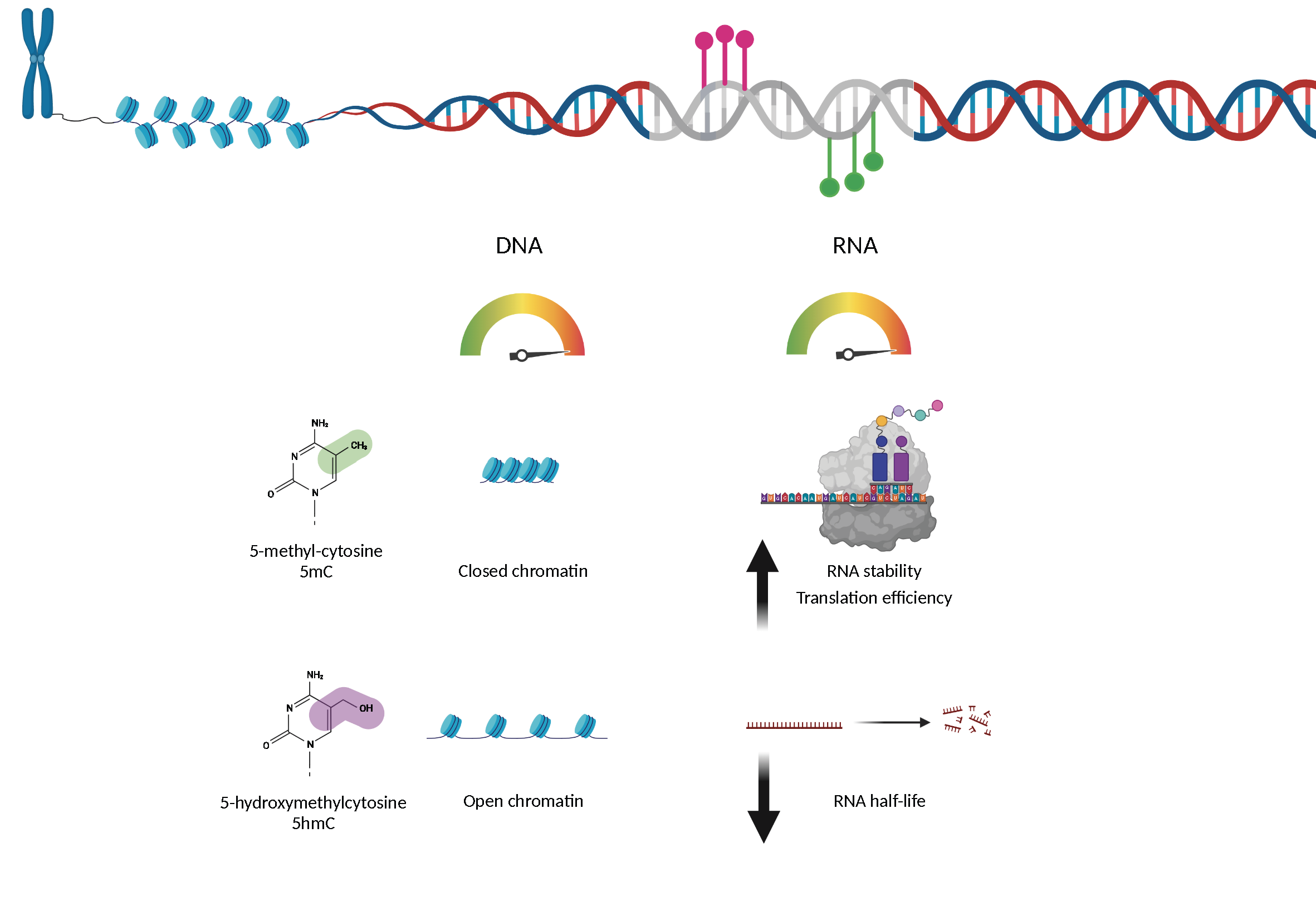

Supplement: Supplementary file 1 [file Image1.TIFF]
